# An Unusual Knee Trauma: Combined Rupture of Medial Collateral Ligament and Patellar Tendon

**DOI:** 10.1155/2014/657296

**Published:** 2014-08-18

**Authors:** T. De Baere, J. De Muylder, A. Deltour

**Affiliations:** Kliniek Sint-Jan, Kruidtuinlaan 32, 1000 Brussel, Belgium

## Abstract

We present the case of a combined lesion of the medial collateral ligament (MCL) and patellar tendon of the knee in a 45-year-old man, after a fall while skiing. Although there are numerous publications concerning associated tears of MCL and other knee ligaments, a combination of MCL tear with a rupture of the patellar tendon is very rare. After a review of the literature and treatment guidelines about these lesions, the clinical case is described and discussed. This knee trauma was treated with a transosseous reinsertion of the patellar tendon, which was reinforced by an allograft of fascia lata, followed by a direct suture of the MCL, which was reinforced with the lateral semitendinosus tendon.

## 1. Introduction

The MCL is the most frequently damaged ligamentous stabilizer of the knee. Injuries to the collateral ligaments are defined into three degrees by the American Medical Association [1]: first degree implies either a microscopic tear or less than one-third of the ligament involved without change in function. Second degree implies a macroscopic tearing with intact fibers remaining (one-third to two-thirds of the ligament involved), giving functional impairment and slight laxity on ligament testing. Third-degree injury implies a complete tear of the ligament with marked instability on ligament testing. Clinically these medial collateral ligament injuries are graded by joint line opening on valgus stress testing with the knee in 30 degrees of flexion, compared to the uninjured knee. An increase in opening of less than 5 mm is a grade I tear, an increase in opening from 5 to 9 mm is a grade II tear, and an increase in opening greater than 10 mm is a grade III tear. These grade III tears imply complete tearing of the ligament [2].

MCL injuries do occur as isolated lesions or in combination with damage to other ligamentous structures (meniscus and/or cruciate ligaments). For instance, Fetto found an 80% incidence of combined ligament injury with grade III MCL tears [3].

There is a general consensus that treatment is nonoperative for grade I and grade II tears of the MCL. For grade III lesions, however, there remains controversy whether treatment should be conservative or surgical. Usually, isolated grade III tears are treated conservatively, but in association with injury to other knee ligaments, there is a tendency to surgically treat the MCL lesion [2, 4, 5]. When surgically treated, usually a reconstruction with a semitendinosus allograft is used, a technique which was originally described by Bosworth [6].

Patellar tendon ruptures usually occur in younger patients as a result of a violent eccentric contraction of the extensor mechanism of the knee. Degenerative processes of the tendon are often present before rupture and numerous authors have indicated a history of pain before rupture [7]. Most of the ruptures occur at the inferior pole of the patella. There is a general consensus that these lesions need surgical treatment and results of surgical treatment are usually favorable [8].

We report the case of a 45-year-old man with a complete tear of the MCL of the knee, combined with a tear of the patellar tendon. In isolation, these lesions appear relatively frequently, but the originality of this case is in the combination of both lesions. We did not find any report of a similar case in the literature. Rae and Davies, in 1991, described a case of a young female with a combined lesion of anterior cruciate ligament, medial collateral ligament, and patellar tendon. They treated this lesion with a suture of the patellar tendon and medial collateral ligament and reported a good functional outcome [9].

## 2. Case Report

A 45-year-old man presented to our clinic with a left knee injury that had occurred a few days before while skiing. He had been immobilized in a brace at the local medical office.

Clinical examination showed marked swelling of the knee joint, with pain at passive mobilization and restricted active motion: 40° of active flexion and an inability to actively extend the knee. Weight-bearing was hardly possible. There was an obvious gap at the level of the insertion of the patellar tendon on the lower pole of the patella. Testing of the MCL compared to the healthy side showed >10 mm widening of the medial joint line with valgus stress in 30° of flexion as well as in full extension. There was no clinical evidence of instability of the other knee ligaments.

**Figure 1:**
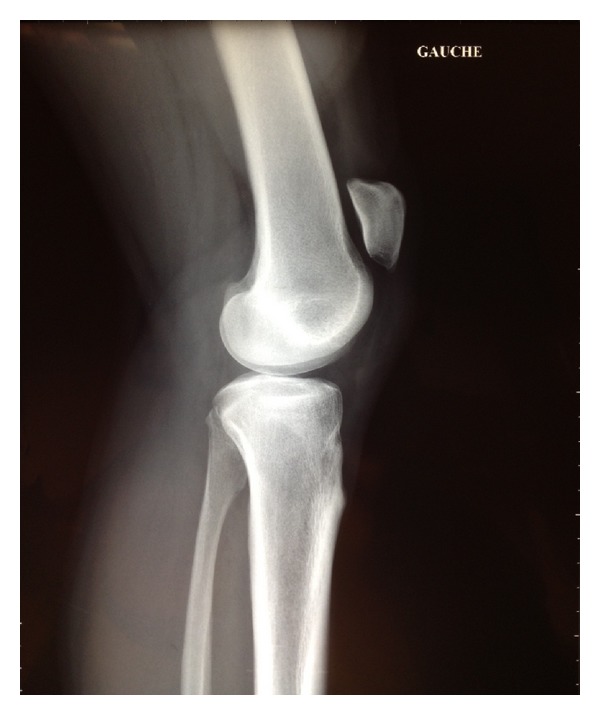


**Figure 2:**
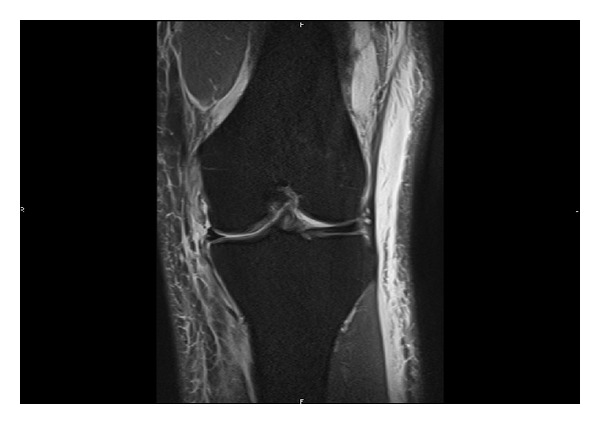


The X-ray of the injured knee showed a superior migration of the patella compared to its usual position (Figure 1). An MRI-scan confirmed the clinical suspicion of a complete tear of the MCL next to its proximal insertion on the medial femoral condyle, as well as a complete rupture of the patellar tendon at the level of its insertion on the lower pole of the patella. There were no lesions of the cruciate ligaments and menisci (Figure 2).

The medical history revealed lower back pain due to a herniated disc, which had been treated conservatively. The patient also reported some pain episodes at the level of the left patellar tendon while jogging in the past. No specific treatment was prescribed for these pains.

Our patient was operated on under epidural anesthesia 5 days after his accident. Clinical examination under anaesthesia confirmed once again the complete instability of the MCL with valgus stress without laxity in the other plains of motion.

At first, we approached the patellar tendon through an anterior longitudinal midline incision. After debridement of the tendinous tissue at the level of the tear, a Krackow-stitch was placed in the patellar tendon distally to its tear. The two loops of this stitch were passed through two bony tunnels in the patella and sutured to each other at the proximal pole of the patella. At the level of the tear, the transosseous reinsertion was reinforced by a running suture of a 3/0 wire. As there was a history of pain at the patellar tendon, we decided to reinforce the reinsertion of the tendon with an allograft of fascia lata, which was sutured directly to the tendinous tissue with absorbable stitches.

The tear of the MCL was approached via an oblique medial incision. At first we performed a direct suture which was reinforced with an autograft of the homolateral semitendinosus tendon. The semitendinosus was isolated with an open stripper, taking care to preserve its distal insertion on the tibia. After suturing it to the MCL, the autograft was fixed proximally with a staple at the level of the medial femoral condyle and distally with a direct suture to its original insertion in order to obtain a double-loop reinforcement. The staple fixation was done in a position of 30° knee flexion and slight varus.

Postoperatively the knee was immobilized in 10° of flexion in a synthetic plaster cast with partial weight-bearing allowed. After 3 weeks the knee was placed in a brace with progressive flexion: 30° the first week, 60° the second week, and 90° the last week. After 6 weeks the brace was removed and complete flexion allowed. A rehabilitation programme with progressive mobilization, proprioceptive training, and muscle strengthening exercises was started.

Clinical control 3 months after the operation showed a limitation of flexion of 20° compared to the other side. There was no swelling of the knee but evident atrophy of the quadriceps muscle without limitation of active extension. Mediolateral stability testing showed no residual valgus instability. A bilateral X-ray of the knee showed normal height of the patella.

At 6 months, full motion was recovered and the patient had returned to normal daily life and recreational sports activities (cycling, fitness). Due to discomfort at the level of the medial femoral condyle, the staple fixing the semitendinosus autograft was removed at 9 months. After this removal, no medial instability occurred. At final follow-up 18 months after the injury, the patient was symptom-free and he had returned to skiing, protecting his knee with a brace.

## 3. Discussion

In the literature, no descriptions of combined lesion of MCL and patellar tendon are found. In contrast, the association of a tear of the MCL, combined with a tear of the anterior cruciate ligament, is well known. In these cases there is a tendency towards surgical treatment of severe MCL lesions.

In a comparative study between conservative and surgical treatment for isolated grade III MCL sprains, Indelicato found comparable results with either treatment [10]. Reider followed 35 patients with an isolated grade III MCL sprain treated with early functional rehabilitation for 5 years and found comparable results to those achieved by surgery in other studies [11]. Jones et al. reported early return to sports in high school football players with isolated grade III MCL injuries treated nonoperatively  [12]. Other researchers confirm these results for as long as there is no associated lesion of the anterior cruciate ligament and results with operative treatment have been shown to be inferior to nonoperative treatment. Stiffness is known to be the most common complication of surgical treatment of isolated grade III MCL sprains [2].

In a literature study, Kovachevich et al.  [13] reviewed the treatment of MCL lesions in dislocated or so-called multiligament injured knees. They conclude that, in these severely traumatized knees, a complete tear of the MCL has a poor healing capacity left on his own and surgical treatment is indicated. Good results are obtained with either direct repair of the MCL or reconstruction with semitendinosus autograft or synthetical graft.

Merritt and Wahl [14] presented a study on the treatment of 138 dislocated and ≪multiligament injured≫ knees. Surgery of these knees is scheduled 2 to 3 weeks after the initial injury, and decision to surgically treat the MCL is made if during surgery an opening 8 to 10 mm is found in the medial compartment with valgus stress in 30° of flexion. These MCL lesions are then treated using the modified Bosworth technique, using the semitendinosus tendon of which the distal insertion is left in place.

Gwathmey et al.   [15] presented the decision algorithm of the Virginia University in multiligament injured knees. If they decide to operate on a torn MCL, their technique is a direct suture, reinforced by a modified Bosworth technique or reconstruction by means of an allograft. In case of the modified Bosworth technique, they prefer harvesting the semitendinosus tendon. In case of an allograft, they also use a semitendinosus.

Futch et al.   [16] reported on a case of a combined anterior cruciate ligament and patellar tendon rupture in a young athlete. This knee was treated with a reconstruction of the anterior cruciate ligament and a suture of the patellar tendon, which was augmented with a patellar tendon allograft. In their opinion, the reinforcement with a patellar tendon allograft biomechanically protects the suture of the tendon through reduction of tension, allowing earlier rehabilitation.

In our case, the combination of a MCL tear with a tear of the patellar tendon is strictly not to be considered as multiligament injured knee. During the surgical intervention that was necessary for the tear of the patellar tendon, the persistent valgus instability justified surgical treatment of the MCL tear. Because our patient already suffered from pain at the level of the patellar tendon before his injury, we decided to use a fascia lata allograft to reinforce the patellar tendon suture. This also allowed for early mobilization.

## 4. Conclusion

To our knowledge, this is the first report of a combined tear of the MCL and the patellar tendon. The surgical treatment, postoperative rehabilitation, and literature on combined knee lesions are discussed.

## References

[1] American Medical Association Committee on the Medical Aspects of Sports (1966). *Standard Nomenclature of Athletic Injuries*.

[2] Bucholz RW (2001). Injuries to the collateral ligaments of the knee. *Rockwood and Green’s Fractures in Adults*.

[3] Fetto JF, Marshall JL (1978). Medial collateral ligament injuries of the knee. *Clinical Orthopaedics and Related Research*.

[4] Miyamoto RG, Bosco JA, Sherman OH (2009). Treatment of medial collateral ligament injuries. *Journal of the American Academy of Orthopaedic Surgeons*.

[5] Terry Canale S (1998). *Campbell’s Operative Orthopaedics*.

[6] Bosworth DM (1952). Transplantation of the semitendinosus for repair of laceration of medial collateral ligament of the knee.. *The Journal of Bone and Joint Surgery A*.

[7] Terry Canale S (1998). Traumatic disorders. *Campbell's Operative Orthopaedics*.

[8] (2001). Patellar tendon rupture. *Rockwood and Green’s Fractures in Adults*.

[9] Rae PJ, Davies DRA (1991). Simultaneous rupture of the ligamentum patellae, medial collateral, and anterior cruciate ligaments: a case report. *American Journal of Sports Medicine*.

[10] Indelicato PA (1983). Non-operative treatment of complete tears of the medial collateral ligament of the knee. *Journal of Bone and Joint Surgery*.

[11] Reider B, Sathy MR, Talkington J, Blyznak N, Kollias S (1994). Treatment of isolated medical collateral ligament injuries in athletes with early functional rehabilitation. A five-year follow-up study. *The American Journal of Sports Medicine*.

[12] Jones RE, Henley MB, Francis P (1986). Nonoperative management of isolated grade III collateral ligament injury in high school football players. *Clinical Orthopaedics and Related Research*.

[13] Kovachevich R, Shah JP, Arens AM, Stuart MJ, Dahm DL, Levy BA (2009). Operative management of the medial collateral ligament in the multi-ligament injured knee: an evidence-based systematic review. *Knee Surgery, Sports Traumatology, Arthroscopy*.

[14] Merritt AL, Wahl CJ (2011). Rationale and treatment of multiple-ligament injured knees: the Seattle perspective. *Operative Techniques in Sports Medicine*.

[15] Gwathmey FW, Shafique DA, Miller MD (2010). Our approach to the management ot the multiple-ligament knee injury. *Operative Techniques in Sports Medicine*.

[16] Futch LA, Garth WP, Folsom GJ, Ogard WK (2007). Acute rupture of the anterior cruciate ligament and patellar tendon in a collegiate athlete. *Arthroscopy*.

